# Gel immersion electrohydraulic lithotripsy for gallbladder stones through a lumen-apposing metal stent

**DOI:** 10.1055/a-2779-5957

**Published:** 2026-01-28

**Authors:** Kiyoyuki Kobayashi, Takako Nomura, Maki Ayaki, Daisuke Namima, Hironobu Suto, Hideki Kamada, Hideki Kobara

**Affiliations:** 1 Department of Gastroenterology and Hepatology, HITO Medical Center, Ehime, Japan; 2 12850 Division of Innovative Medicine for Hepatobiliary and Pancreatology, Faculty of Medicine, Kagawa University, Kagawa, Japan; 3 38078 Department of Gastroenterology and Neurology, Faculty of Medicine, Kagawa University, Kagawa, Japan; 4 38078 Department of Gastroenterological Surgery, Faculty of Medicine, Kagawa University, Kagawa, Japan

Peroral cholecystoscopy through a lumen-apposing metal stent (LAMS) after endoscopic ultrasound-guided gallbladder drainage (EUS-GBD) enables the direct visualization and lithotripsy of gallbladder stones 1 2 . However, saline irrigation may fail to achieve complete stone submersion because of rapid outflow through the large-caliber LAMS 3 . Gel immersion endoscopy maintains a clear visual field with lower intraluminal pressure than saline 4 and has been applied for bile duct lithotripsy 5 , but not yet reported for gallbladder stones.

A 78-year-old woman, a poor surgical candidate, presented with acute cholecystitis caused by a 15-mm stone impacted in the gallbladder neck ( Fig. 1 ). EUS-GBD was performed using a 10-mm Hot AXIOS stent (Boston Scientific, Marlborough, MA, USA; Fig. 2 ). Acute cholecystitis resolved promptly.

**Fig. 1:**
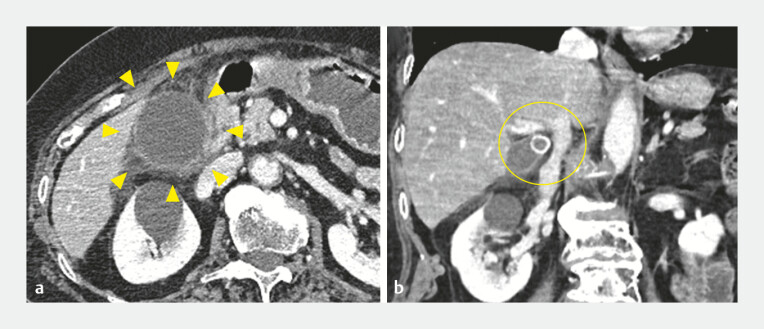
Contrast-enhanced computed tomography at the time of diagnosis of acute cholecystitis. **a** An axial view showing gallbladder distension, wall thickening, and pericholecystic inflammation (yellow arrowheads). **b** A coronal view demonstrating a 15-mm stone impacted in the gallbladder neck (yellow circle).

**Fig. 2:**
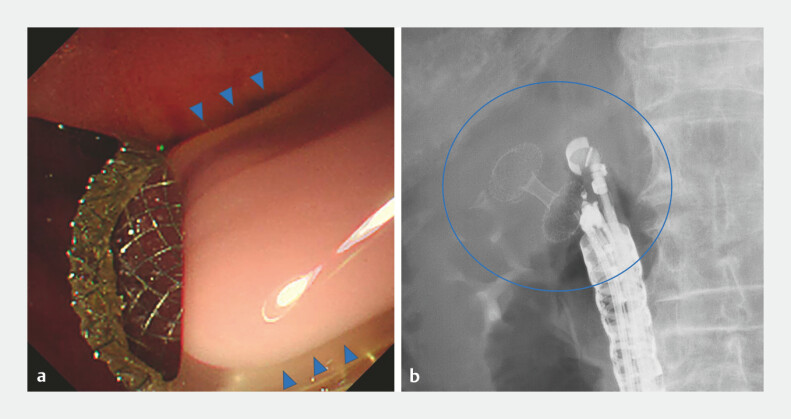
Endoscopic ultrasound-guided gallbladder drainage (EUS-GBD) for acute cholecystitis. **a** An endoscopic view after lumen-apposing metal stent (LAMS) deployment, showing drainage of purulent bile (blue arrowheads). **b** A fluoroscopic image confirming the placement of the LAMS from the duodenal bulb to the gallbladder (blue circle).

Ten days later, peroral cholecystoscopy was performed using an ultrathin endoscope (GIF-1200N; Olympus, Tokyo, Japan). Gel immersion electrohydraulic lithotripsy (EHL) was performed using Viscoclear (Otsuka Pharmaceutical Factory, Tokushima, Japan), an electrolyte-free gel with viscoelastic properties compatible with electrosurgical procedures 4 . The gel was injected through the working channel. Despite the large-caliber LAMS, 10 mL per injection sufficed to submerge the stone and provide excellent visualization during EHL ( Fig. 3 ). The viscosity minimized fragment dispersion and maintained a clear field ( Video 1 ). The gel was easily removed by aspiration and saline flushing. Complete stone clearance was achieved using a Memory Basket Eight Wire (Cook Medical, Bloomington, IN, USA), with a total gel volume of less than 100 mL ( Fig. 4 ). In larger gallbladders, excess gel would naturally drain through the LAMS. Other gels with similar properties may be applicable.

**Fig. 3:**
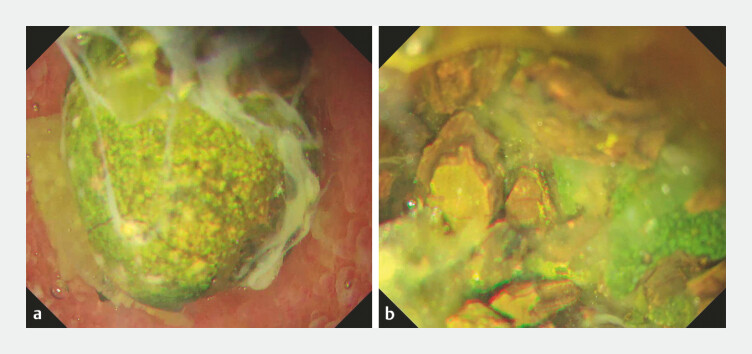
Endoscopic views during gel immersion electrohydraulic lithotripsy (EHL) via the lumen-apposing metal stent (LAMS) using an ultrathin endoscope. **a** Complete stone submersion is achieved with a small volume of gel despite the large-caliber LAMS. **b** Stone fragmentation is performed using EHL.

Gel immersion electrohydraulic lithotripsy for gallbladder stones through a lumen-apposing metal stent.Video 1

**Fig. 4:**
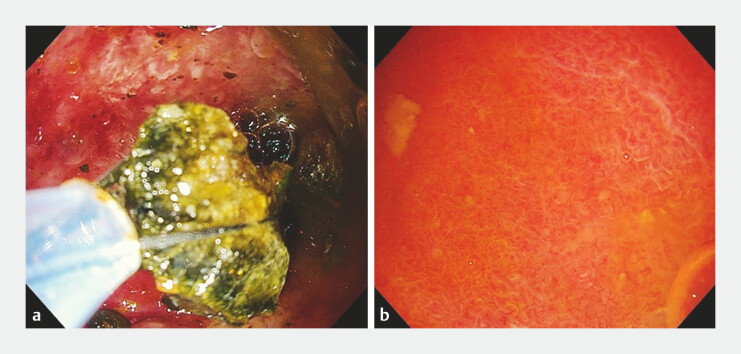
Endoscopic views during stone extraction and final inspection under gel immersion. **a** Stone fragments are retrieved using an 8-wire basket. **b** Final cholecystoscopy under gel immersion confirms complete stone clearance.

The low-pressure environment provided by gel immersion was considered advantageous for the fragile gallbladder wall following acute inflammation. The patient recovered without complications.

This is the first report of gel immersion EHL through a LAMS for gallbladder stone removal, effectively overcoming the limitations of saline irrigation.

Endoscopy_UCTN_Code_CCL_1AZ_2AD

Endoscopy_UCTN_Code_CPL_1AK_2AF

Endoscopy_UCTN_Code_TTT_1AR_2AH

Endoscopy_UCTN_Code_TTT_1AR_2AL

## References

[1] Chan SM Teoh AYB Yip HC Feasibility of per-oral cholecystoscopy and advanced gallbladder interventions after EUS-guided gallbladder stenting (with video) Gastrointest Endosc 2017 85 1225 1232 10.1016/j.gie.2016.10.014 27756612

[2] Campbell C Pawa R Definitive nonsurgical management of stump cholecystitis with EUS-guided lumen-apposing metal stent placement and electrohydraulic lithotripsy VideoGIE 2023 8 203 205 10.1016/j.vgie.2023.01.008 37197168 PMC10183644

[3] Vara-Luiz F Mendes I Nunes G Endoscopic ultrasound-guided cholecystoduodenostomy followed by stone clearance using electrohydraulic and mechanical lithotripsy in a frail patient with acute cholecystitis Endoscopy 2024 56 E1006 E1007 10.1055/a-2462-0975 39557066 PMC11573452

[4] Yano T Takezawa T Hashimoto K Gel immersion endoscopy: Innovation in securing the visual field – Clinical experience with 265 consecutive procedures Endosc Int Open 2021 9 E1123 E1127 10.1055/a-1400-8289 34222638 PMC8216780

[5] Miyano A Ogura T Okuda A Gel-immersion electrohydraulic lithotripsy during digital single-operator cholangioscopy is helpful when bleeding occurs Endoscopy 2023 55 E98 E99 10.1055/a-1941-8488 36216256 PMC9829775

